# Ubiquitin-Specific Protease 21 Promotes Colorectal Cancer Metastasis by Acting as a Fra-1 Deubiquitinase

**DOI:** 10.3390/cancers12010207

**Published:** 2020-01-14

**Authors:** Sun-Il Yun, Hye Kyung Hong, So-Young Yeo, Seok-Hyung Kim, Yong Beom Cho, Kyeong Kyu Kim

**Affiliations:** 1 Department of Precision Medicine, Sungkyunkwan University School of Medicine, Suwon 16419, Korea; yunsi@skku.edu; 2 Department of Surgery, Samsung Medical Center, Sungkyunkwan University School of Medicine, Seoul 06531, Korea; hyekyung@samsung.com; 3 Department of Pathology, Samsung Medical Center, Sungkyunkwan University School of Medicine, Seoul 06531, Korea; soyoung433@skku.edu; 4 Samsung Medical Center, Department of Health Science and Technology, Samsung Advanced Institute for Health Science and Technology, Sungkyunkwan University School of Medicine, Seoul 06531, Korea

**Keywords:** deubiquitination, USP21, Fra-1, colorectal cancer, metastasis

## Abstract

Fos-related-antigen-1 (Fra-1), a member of the activator protein-1 (AP-1) transcription factor superfamily, has an essential role in cancer progress and metastasis and Fra-1 is considered a therapeutic target in metastatic cancer including metastatic colorectal cancer (mCRC). However, its regulation at protein level has not yet been clearly elucidated. We found that ubiquitin-specific protease 21 (USP21) increases Fra-1 stability by deubiquitinating Fra-1 and enhances the expression of Fra-1 target genes in colon cancer cells. We also showed that USP21 controlled Fra-1-dependent migration and invasion activities. The oncogenic property of USP21 was confirmed by a significant reduction in liver metastasis when USP21-knockdown cancer cells were injected intrasplenically into mice. Consistently, clinicopathological analysis of colorectal cancer patients revealed a correlation of USP21 expression with high-grade carcinoma and life span. These results demonstrate that USP21 enhances Fra-1 stability and AP-1 target gene expression by deubiquitinating Fra-1. Therefore, USP21 is considered an attractive therapeutic target in mCRC with high Fra-1 expression.

## 1. Introduction

Due to the advances in screening techniques and targeted cancer therapy, the death rate from colorectal cancer (CRC) has been decreasing. However, CRC is the second leading cause of cancer death in the world, with a poor five-year survival for patients diagnosed with metastatic colorectal cancer (mCRC) [1]. Therefore, a targeted therapy for mCRC is necessary for effective treatment of CRC to improve survival rate. 

Activator protein 1 (AP-1), a key transcription factor controlling differentiation, proliferation, and apoptosis, is composed of members of Fos (c-Fos, FosB, Fos-related-antigen-1 (Fra-1), and Fra-2) and Jun (c-Jun, JunB, and JunD) protein families. Jun family proteins can homodimerize, but Fos family proteins must form heterodimers with Jun family proteins for full activity [2]. Due to the role of AP-1 in multiple key signaling pathways, dysregulated expression of AP-1 proteins is closely linked to tumorigenesis [3,4]. Fos-related antigen-1 (Fra-1), a Fos family protein involved in regulating cell proliferation, differentiation, and transformation by dimerizing with Jun family proteins to form AP-1, is highly expressed in human colon [5], breast [6], lung [7], esophagus [8], and bladder cancers [9]. In addition, Fra-1 contributes to cell motility and invasion [10,11], which are necessary for metastasis. Implication of Fra-1 involvement in cancer metastasis is evident through its ability to induce epithelial–mesenchymal transition (EMT) by increasing the expression level of EMT-related genes such as matrix metallopeptidases (MMPs) [12]. Especially, Fra-1 increases MMP-1 expression through direct binding to the AP-1 site in the MMP-1 promoter [13].

In CRC, Fra-1 is strongly expressed at the invasive front of budding tumor cells, and epithelial–mesenchymal programming during cancer progression is under the control of Fra-1-mediated transcriptional activity [12]. Further, KRAS gene is frequently mutated in human cancers, and CRC harboring KRAS mutation induces continued oncogenic KRAS expression for tumor maintenance. The canonical ERK pathway normally promotes AP-1 activity and activated RAS-ERK signaling induces transcriptional expression of Fra-1 [14,15] to protect against proteasomal degradation by post-transcriptional stabilization [16]. This suggests that Fra-1-mediated transcriptional activity is regulated at the protein level [11]. However, the ubiquitin-dependent protein stability of Fra-1 has not been as well studied as that of c-Jun, the representative heterodimeric partner of Fra-1 [17]. Since the AP-1 complex has a key role in many cancers, its transcriptional activity is likely to be controlled at the protein level by ubiquitination and deubiquitination.

One recent approach for cancer treatment is to remove oncogenic proteins by controlling the ubiquitin-proteasome system (UPS) [18], which plays a major role in protein degradation. K48-linked ubiquitination mediated by Ub-activating enzyme (E1), Ub-conjugating enzyme (E2), and Ub-ligases (E3) directs target proteins to the proteasome for degradation. As a negative feedback mechanism, Ub-chains can be removed from substrates by deubiquitinating enzymes (DUBs) to increase the stability of the substrate proteins. DUBs, including ubiquitin-specific proteases (USPs), play a key role in cancer formation and progression in various tumors by increasing the stability of oncogenes. For example, the canonical Wnt-signaling pathway is an important signaling pathway in colorectal cancer. USP14 has an oncogenic role by enhancing Wnt signaling through deubiquitinating Dishevelled (Dvl), a key regulator of Wnt signaling [19]. USP4 and USP7 also play a role in tumor progression by enhancing the stability of β-catenin, which is involved in Wnt signaling [20,21,22,23]. USP22 is also critical to CRC stemness and chemoresistance through the Wnt/β-catenin pathway [24].

Further, activation of Wnt/β-catenin signaling directly activates transcription of Fra-1 [25], which regulates canonical Wnt signaling by modulating the expression of Wnt pathway components and transcriptional activity of β-catenin in cancer cells [26]. Based on the role of Fra-1 in cancer metastasis including mCRC, it is proposed that removing Fra-1 by inhibiting Fra-1 DUBs can be a promising strategy for mCRC treatment, since many Fra-1 target genes relevant to cancer metastasis can be downregulated simultaneously by removing or decreasing the stability of Fra-1.

Previously, we screened for DUBs that show strong deubiquitination activity in 293T cells by monitoring the amount of total ubiquitinated protein after transfection with DUB-encoding plasmids [20]. This process led to the identification of 20 DUBs that belong to the USP subfamily. Among the tested DUBs, we newly identified USP21 to play an oncogenic role in CRC as a Fra-1 DUB and to regulate a Fra-1 target gene involved in cancer metastasis. In addition, the present study shows that USP21 knockdown decreases tumor formation and cancer metastasis in vivo using a mouse model, and USP21 expression is upregulated in colorectal cancer patients. In the end, we propose USP21 downregulation as a potential therapeutic strategy for mCRC.

## 2. Results

### 2.1. USP21 Binds and Regulates Fra-1 Expression

Based on the assumption that DUBs enhance the transcriptional activity of AP-1 by deubiquitinating Fra-1, a main component of the AP-1 complex, we searched for DUBs that enhanced AP-1 luciferase reporter activity in 293T cells. To identify the Fra-1 DUB, we co-transfected DUB-encoding plasmids with Fra-1 and AP-1 luciferase reporters into 293T cells and monitored AP-1 transcriptional activity by luciferase assay and Fra-1 expression by immunoblotting. This process led to the identification of USP21 that resulted in the greatest enhancement of AP-1 transcriptional activity and Fra-1 expression (Figure S1). Therefore, we assumed that USP21 is a Fra-1 DUB and further investigated its role in regulating Fra-1 expression.

**Figure 1:**
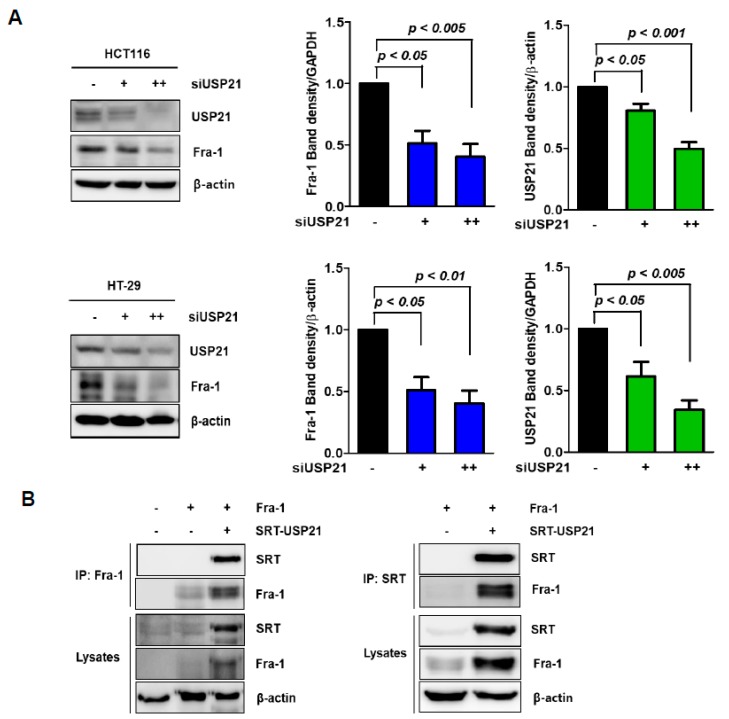
Ubiquitin-specific protease (USP)21 interacts with Fra-1. (**A**) HCT116 and HT-29 cells were transfected with scrambled siRNA (−: negative) or siUSP21 (+: 30 nM, ++: 60 nM) for 48 h, and then the expression of Fos-related-antigen-1 (Fra-1) and USP21 was examined with total cell lysates by immunoblotting. Quantification of Fra-1 band intensities is shown on the right. The data were statistically analyzed by one-way ANOVA (*n* = 3). (**B**) 293T cells were co-transfected with plasmids encoding Fra-1 with or without SRT tagged USP21 for 24 h, and then the cells were harvested. The cell lysates were immunoprecipitated with anti-Fra-1 or anti-SRT antibody and then subsequently immunoblotted with anti-SRT and anti-Fra-1 antibodies as indicated. Total cell lysates (lysates) were also analyzed, and expression of β-actin was used as a loading control.

To investigate the molecular mechanism by which USP21 affects AP-1 transcriptional activity, the protein expression level of Fra-1 was quantitated in colon cancers. First, we determined the effect of USP21 on stabilizing Fra-1 under USP21 knockdown conditions in two colon cancer cell lines, HCT116 and HT-29. siUSP21 significantly decreased Fra-1 protein in a dose-dependent manner (Figure 1A and Figure S2).

To further investigate whether USP21 interacts with Fra-1, we performed immunoprecipitation in 293T cells (Figure 1B). This result showed a reciprocal interaction between USP21 and Fra-1.

### 2.2. Fra-1 Protein is Degraded by Proteasome

**Figure 2:**
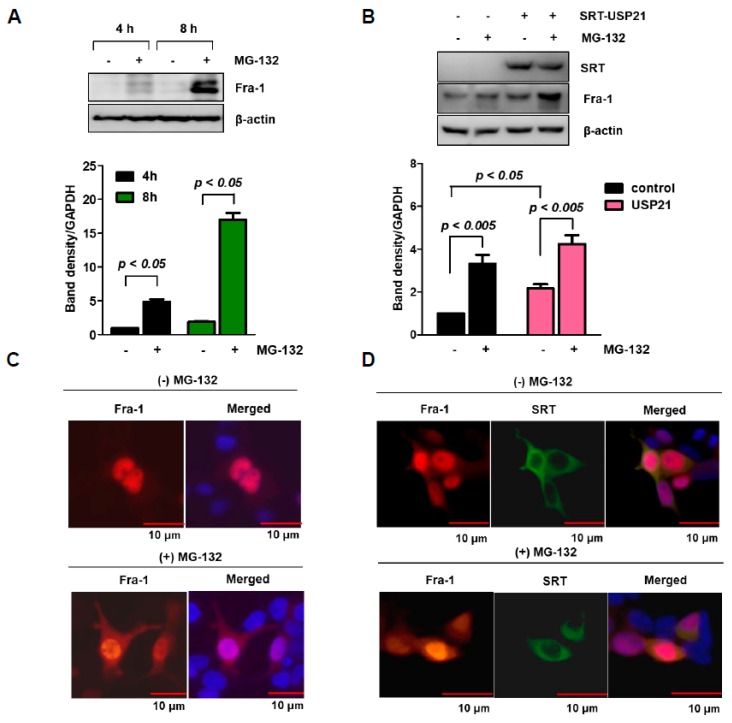
Fra-1 protein is degraded by proteasomes. (**A**) HCT116 cells were treated with or without MG-132 for 4 h and 8 h, and then endogenous Fra-1 protein expression was examined by Western blotting. Band intensities were quantified as shown in the bottom panel. (**B**) HCT116 cells were transfected with SRT-USP21 for 24 h and then treated with or without MG-132 for 6 h before cell harvesting. Total cell lysates were analyzed by Western blotting. Quantification of band intensities was statistically analyzed by one-way ANOVA (*n* = 3). (**C**) 293T cells were transfected with plasmid encoding Fra-1, and immunostaining for Fra-1 was performed in the absence or presence of MG-132 treatment for 6 h. Images of Fra-1 (red) and nuclei (blue) were obtained with a confocal fluorescent microscope (scale bar = 10 μm). (**D**) 293T cells were co-transfected with Fra-1 and SRT-USP21 plasmids for 24 h and were then treated with MG-132 for 6 h before cell fixation. The cells were stained with anti-Fra-1 (red) and anti-SRT (green) antibodies, respectively. Nuclei (blue) were stained with DAPI, and a merged cell image of the three colors is shown (scale bar = 10 μm).

To determine whether Fra-1 stability is negatively controlled by the ubiquitination-dependent proteasomal pathway, we examined protein degradation of Fra-1 in HCT116 cells after treatment with MG-132, a proteasome inhibitor. Analysis of total cell lysates showed time-dependent, continuous accumulation of Fra-1 protein (Figure 2A). In addition, to investigate whether Fra-1 stability is positively regulated by DUB-dependent deubiquitination, a plasmid encoding USP21 was transfected into HCT16 cells. Ectopic expression of USP21 further increased Fra-1 expression compared to cells lacking MG-132 treatment (Figure 2B).

To examine the effects of MG-132 and USP21 on Fra-1, we next observed the cellular distribution of Fra-1 protein by immunostaining. Fra-1 was present in the nucleus but was also distributed in the cytosol after MG-132 treatment (Figure 2C). Contrary to Fra-1, USP21 protein appeared in the cytosol, where some amount of Fra-1 was co-localized USP21 with or without MG-132 treatment (Figure 2D).

### 2.3. USP21 Functions as a Fra-1 DUB

**Figure 3:**
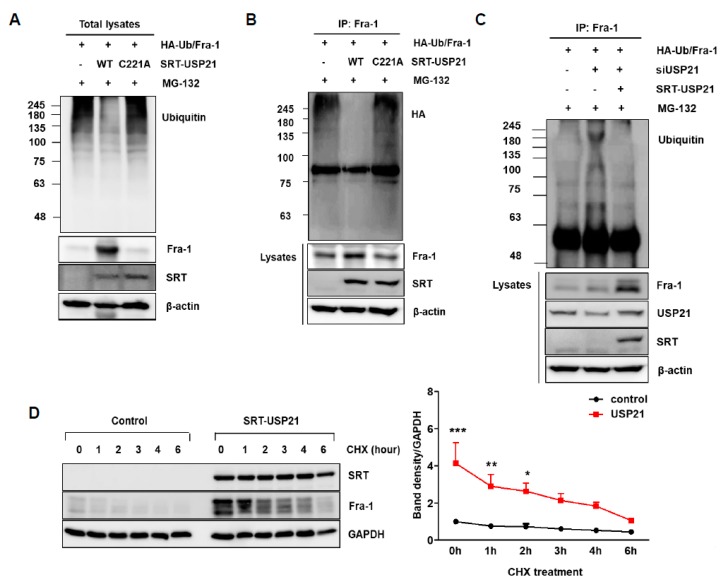
USP21 regulates degradation of Fra-1 protein. (**A**–**C**) To determine Fra-1 ubiquitination, Fra-1 plasmid was co-transfected with HA-Ub and SRT-USP21 wild type (WT) or SRT-USP21 C221A in 293T cells. The total cell lysates were immunoblotted with the indicated antibodies (**A**) and followed by immunoprecipitation (IP) with anti-Fra-1 antibody (**B**). Ubiquitinated Fra-1 level was determined by immunoblotting with anti-ubiquitin or anti-HA antibody before and after IP. Expression of β-actin was used as a loading control. (**C**) After transfection with siUSP21 for 24 h, 293T cells were co-transfected with plasmids encoding HA-Ub, Fra-1, and SRT-USP21 WT and treated with MG-132 for 6 h before cell harvesting. The cell lysates were immunoprecipitated with anti-Fra-1 antibody and subsequently immunoblotted with anti-HA antibody. Total cell lysates were immunoblotted with indicated antibodies, and expression of β-actin was used as a loading control. (**D**) HCT116 cells were transfected with empty vector (control) and SRT-USP21 for 24 h, after which cycloheximide (CHX) was applied for the indicated times. The expression levels of Fra-1, USP21, and GAPDH were measured by immunoblotting, and quantification of band intensities from triplicate determinations is shown at the bottom. Significance was determined using two-way ANOVA test between two groups (*** *p* < 0.001, ** *p* < 0.01, * *p* < 0.05).

To investigate whether the catalytic activity of USP21 affects Fra-1 stability, a plasmid construct encoding mutant USP21 (C221A) that lacked catalytic activity due to a Cys-to-Ala substitution at 221 was constructed. Next, to determine whether USP21 facilitates deubiquitination of Fra-1 through its enzymatic activity, we examined the extent of ubiquitination (Ub) in total cell lysates (Figure 3A) and Fra-1-immunoprecipitation (IP) samples (Figure 3B) from 293T cells transfected with plasmids encoding wild type (WT) or mutant (C221A) USP21. Overexpression of WT USP21 resulted in the reduction of ubiquitinated proteins in the total cell lysate (Figure 3A) and complete removal of Ub from Fra-1 (Figure 3B). However, overexpression of C221A USP21 did not change ubiquitination levels (Figure 3A,B). Consistent with this observation, reduction in USP21 expression by siRNA promoted ubiquitination of Fra-1, which was reversed by the addition of WT USP21 (Figure 3C). These results demonstrate that Fra-1 is a substrate of USP21, and that its stability is controlled by ubiquitination. Consistently, USP21 overexpression in HCT116 cells clearly increases the half-life of Fra-1 protein in the presence of cycloheximide (CHX) (Figure 3D). Based on this result, this study confirmed that USP21 is required for protein stability of Fra-1 in colon cancer cells. Therefore, this study suggests that USP21 is a DUB of Fra-1.

### 2.4. USP21 Regulates Fra-1-Mediated Transcriptional Activity 

**Figure 4:**
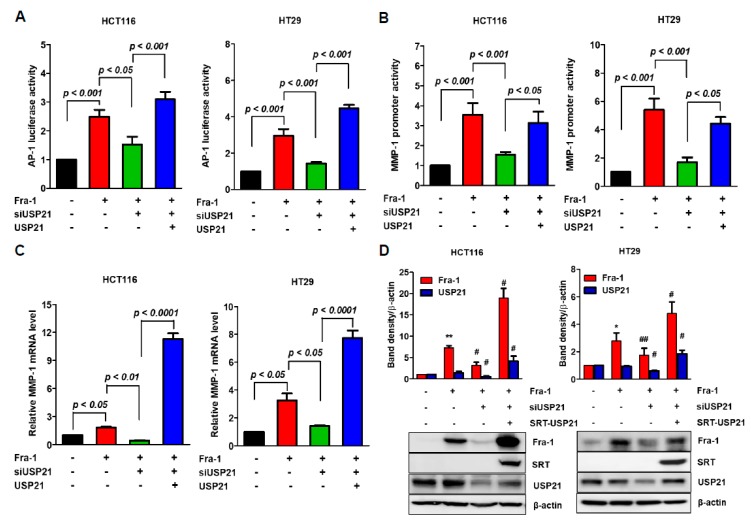
USP21 regulates Fra-1 activities. (**A**,**B**) HCT116 and HT-29 cells were transfected with scrambled siRNA (control) or siUSP21 for 24 h and then co-transfected with plasmids encoding Fra-1 or SRT-USP21 as indicated. After introducing AP-1 (A) or MMP-1 reporter plasmid (B) for an additional 24 h, luciferase transcriptional responses were measured in each cell line. Data were statistically analyzed by one-way ANOVA (*n* = 3). (**C**) After transfection with indicated plasmids into HCT116 and HT-29 cells, MMP-1 mRNA expression was quantified by real time-PCR with normalization to GAPDH. Values are fold-increases relative to the control, and error bars represent mean ± SD. Data were statistically analyzed by one-way ANOVA (*n* = 4). (**D**) HCT116 and HT-29 cells were transfected with scrambled siRNA (control) or siUSP21 for 24 h and subsequently co-transfected with plasmids encoding Fra-1 or SRT-USP21. Total cell lysates were analyzed by immunoblot analysis with the indicated antibodies. Quantification of band intensities was also shown by graph (*n* = 3–4). ** *p* < 0.01 vs. control, ## *p* < 0.01 vs. Fra-1, and # *p* < 0.05 vs. Fra-1.

We examined the effects of USP21 on Fra-1-dependent AP-1 transcriptional activity using luciferase reporters in colon cancer cells (Figure 4A). Contrary to the effect of WT USP21 overexpression, USP21 knockdown significantly decreased AP-1 transcriptional activity. However, complementation of WT USP21 to USP21 knockdown cells resulted in the rescue of AP-1 transcriptional activity (Figure 4A).

To ensure that these findings held true for colon cancer cells (HCT116), we investigated the effect of USP21 on Fra-1 activity by measuring the expression of MMP-1, a target gene of Fra-1 as AP-1 binds to the MMP-1 promoter [27]. We measured the effect of USP21 on Fra-1 activity by estimating MMP-1 promoter luciferase assay after Fra-1 transfection followed by USP21 knockdown. As shown in Figure 4B, USP21 knockdown clearly reduced luciferase activity, suggesting that USP21 knockdown reduced Fra-1 activity. Due to confirming the enhanced Fra-1 transcriptional activity by USP21, we also measured MMP-1 mRNA expression by quantitative real-time PCR analysis under the same conditions (Figure 4C). When Fra-1 induced MMP-1 expression, USP21 knockdown reduced the Fra-1 effect on MMP-1 expression. Reversely, the additional overexpression of USP21 in USP21 knockdown cells highly enhanced MMP-1 expression (Figure 4C). Under these experimental conditions, siUSP21 decreased protein expression of Fra-1 as determined by immunoblotting but transfection with the USP21 gene recovered the Fra-1 expression (Figure 4D). To compare the difference between WT and mutant (C221A) USP21, Fra-1-mediated transcriptional activity was analyzed. Expectedly, mutant USP21 did not increase AP-1 luciferase activity (Figure S3A). Consistent with the AP-1 luciferase reporter assay results, we found that mutant USP21 had no effect on MMP-1 promoter activity (Figure S3B) or mRNA level of MMP-1 (Figure S3C). Together, these results confirmed that Fra-1 protein level was enhanced under these conditions, and the catalytic activity of USP21 stabilized Fra-1 protein (Figure S3D). However, there was no significant difference in Fra-1 gene expression by USP21 overexpression (Figure S4). Based on these results, this present study indicates that USP21 promotes Fra-1-mediated MMP-1 expression by increasing the expression level of Fra-1 protein.

### 2.5. USP21 Is Associated with Invasion and Migration Activity of Colon Cancer Cells

**Figure 5:**
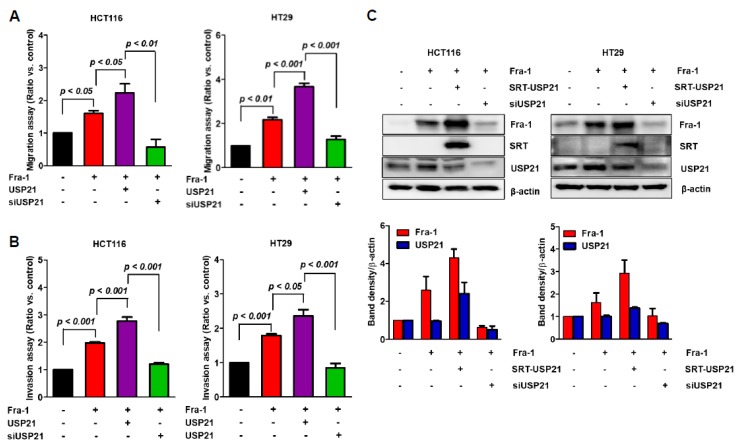
USP21 is related to cancer cell activity. (**A**–**C**) HCT116 and HT-29 cells were co-transfected with or without SRT-USP21 and Fra-1 plasmids for 24 h. Fra-1 plasmid was co-transfected with or without USP21 knockdown in each cell line. Transfected cells were subjected to (**A**) migration assays for 24 h and (**B**) invasion assays for 48–72 h. Data shown are average values from three independent experiments. Data were statistically analyzed by one-way ANOVA (*n* = 3). (**C**) Total cell lysates were analyzed by immunoblot analysis with the indicated antibodies. Band intensities of Fra-1 and USP21 were quantified as shown in the graph below (*n* = 2).

The expression level of Fra-1 in colon cancer is strongly correlated with cell migration and invasion [28]. We therefore evaluated the effect of USP21 on both migration (Figure 5A) and invasion activities (Figure 5B) in colon cancer cells, HCT116 and HT29. First, we verified that Fra-1 expression enhanced migration activity (Figure 5A). Then, we compared the migration activity of Fra-1 to that of co-overexpressing Fra-1 with USP21 or siUSP21. We found that USP21 significantly increased the migration activity of cancer cells, and USP21-knockdown reversely reduced cell migration. Consistently, the invasion activity of cancer cells was significantly induced by USP21, while USP21 knockdown reduced the Fra-1 effect (Figure 5B). It was also confirmed that the Fra-1 level was also proportional to the migration and invasion activity (Figure 5C). These results confirmed that USP21 is associated with the invasion and migration of colon cancer cells by controlling Fra-1 activity.

### 2.6. USP21 Expression Is Upregulated in Colorectal Cancer Tissues and Associated with a Poor Prognosis

**Figure 6:**
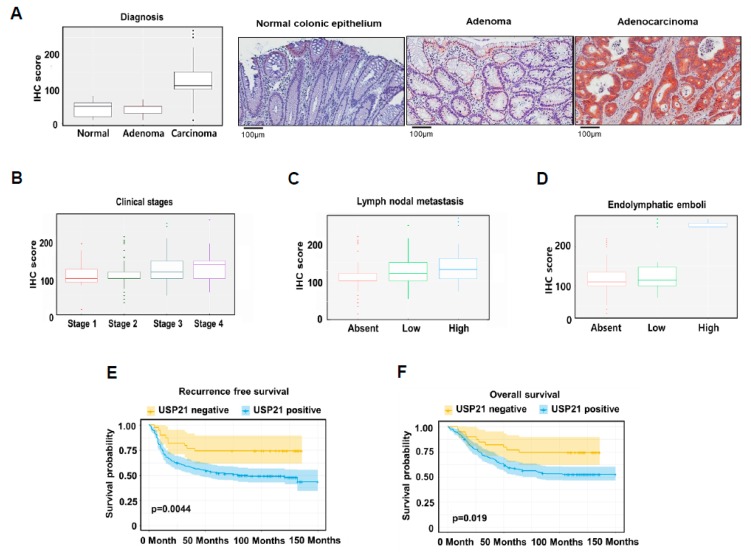
USP21 expression is highly correlated with cancer progression in colon cancer patients. (**A**,**B**) The expression of USP21 protein following the Oncotype DX assay (A, left) and according to tumor stage in CRC patients (B). Data were statistically analyzed by SPSS (version 18.0). Representative IHC images of USP21 expression in CRC patient samples (A, right). (**C**,**D**) Correlation of USP21 expression with nodal metastasis of CRC (C) and with endolymphatic emboli of CRC (D). Chi-squared tests (Pearson’s Chi-squared test or Chi-squared test using linear by linear association) were used to analyze correlations between IHC results and clinicopathologic parameters. (**E**,**F**) Kaplan–Meier survival curves of CRC patients: recurrence-free survival (E) and overall survival (F). Kaplan–Meier survival curves were drawn using the survival probability of CRC patients in all stages.

To further corroborate the role of USP21 in colorectal cancer, the expression of USP21 was examined in 297 colorectal cancer (CRC) patient samples using immunohistochemistry and was analyzed for correlation with clinicopathological parameters (Table 1). Expression of USP21 in patient tumor samples was represented by immunohistochemistry (IHC) score, which was obtained by multiplying the percentage of USP21-positive cells by staining intensity. For example, if all cancer cells stained at maximal intensity (3+), the IHC score was 300 (100% × 3). USP21 expression was found to be significantly enhanced in colorectal adenocarcinoma cells compared to normal cells and adenoma cells (Figure 6A left, Table 1). The IHC images of USP21 expression in CRC patient samples also illustrate the correlation between USP21 expression and cancer stage (Figure 6A, right). In addition, USP21 level was significantly increased in stage 4 colorectal cancer compared to earlier stages (Stages 1 and 2) (Figure 6B, Table 1). Interestingly, USP21 expression was significantly enriched in colorectal cancers with frequent lymph node metastasis (N2b, number of cancer cell-positive lymph nodes ≥4, Figure 6C) and high number of endolymphatic tumor emboli (Figure 6D). In agreement with these findings, expression of USP21 in colorectal cancer cells was significantly correlated with a poor prognosis (recurrence-free survival, *p* = 0.0044, Figure 6E, Table S1; overall survival, *p* = 0.019, Figure 6F, Table S2). These results support an oncogenic role of USP21 in the development of colorectal cancer as well as cancer metastasis.

### 2.7. Effect of USP21 on In Vivo Metastasis 

**Figure 7:**
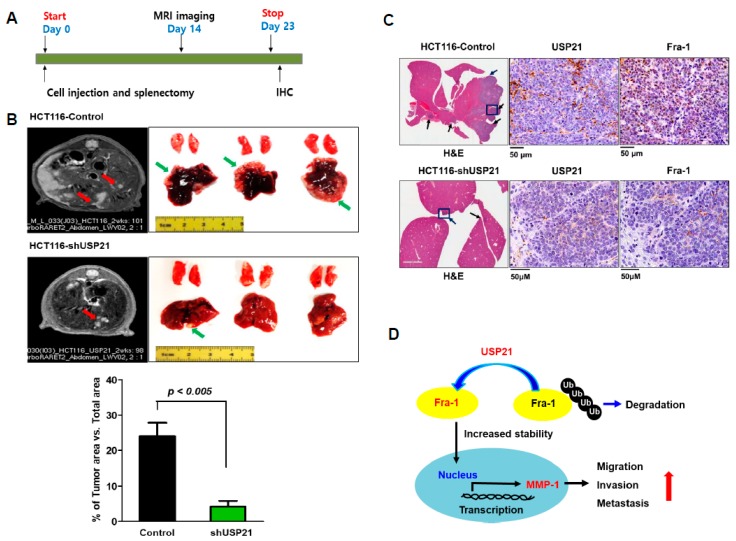
USP21 knockdown in colon cancer decreases in vivo metastasis. (**A**) The scheme of designed animal experiments. (**B**) Representative magnetic resonance images (MRIs) of tumors from HCT116-control and HCT116-USP21 knockdown groups at day 14 after intrasplenic injection (left). Developed tumors are marked by red arrows. Representative morphology of tumor formation in mouse liver driven by both HCT116-control and HCT116-USP21 knockdown at day 23 after intrasplenic injection (right). Tumor regions are indicated by green arrows. The extent of tumor formation was quantified as percentage ratio (%) of tumor-forming area to total area of the liver. Data were statically analyzed by *t*-test (HCT116-control, *n* = 6 and HCT116-shUSP21, *n* = 4). (**C**) Before applying the primary antibody, metastatic tumors in the livers of mice in HCT116-control and HCT116-USP21 knockdown groups was stained with hematoxylin and eosin (H&E). Black arrows show tumors, and blue rectangles indicate the region of immunohistochemistry from a representative tumor sample. Positive expression of USP21 and Fra-1 was detected (scale bar = 50 μm). (**D**) A schematic model for the role of USP21 in colorectal cancer metastasis. USP21 deubiquitinates Fra-1 to increase transcription of MMP-1, an Fra-1 target gene, and promotes cell motility and metastasis.

Since this study showed that USP21 was associated with enhanced migration and invasion activities in colon cancer, we tested the effect of USP21 on the metastatic ability of cancer cells in a mouse model (Figure 7A). The control group (HCT116-control) comprised six nude mice into which HCT116 cells (3.5 × 10^6^) were injected intrasplenically, followed by splenectomy (Table 2). In the same way, USP21-knockdown HCT116 cells were created by puromycin selection after shUSP21 transfection into HCT116 cells (Figure S5) and then were injected into the experimental group (HCT116-shUSP21). Magnetic resonance imaging (MRI) monitored the rate of liver metastasis in each group every week and liver metastasis in the control group was confirmed on day 14 after injection (Figure 7B, left). Mice were sacrificed on day 23 and their tumors characterized (Figure 7B, right). In both groups, metastatic tumor was found in the liver. However, tumors in the experimental group of USP21 knockdown were smaller and less numerous as presented by the percentage of the tumor forming area: 4.2% in the USP21 knockdown group and 24% in the control group (Figure 7B, bottom). This result is consistent with reduced migration and invasion activity of USP21-knockdown cells (Figure 5). These results suggest that USP21 contributes to migration and metastasis of cancer cells. To further confirm the contribution of USP21 to tumor progression, expression levels of USP21 and Fra-1 in tumor tissues from control and experimental groups were examined by immunohistochemistry. This analysis revealed that Fra-1 level was significantly reduced in the HCT116-shUSP21 experimental group relative to the control group (Figure 7C). These results indicate that elevated expression of USP21 is associated with colon cancer metastasis.

## 3. Discussion

Fra-1 is a representative prognostic marker of metastasis in various cancers [7,10,29]. Particularly, Fra-1 is highly abundant in human colon cancers [5,26], and aberrant expression of Fra-1 is correlated with EMT of mCRC [6,30]. Since Fra-1 regulates the expression of many genes involved in tumor invasion, especially MMP-1 expression in breast [31], osteosarcoma [32], and colon cancers [33], targeting Fra-1 seems to be a promising strategy for treatment of mCRC. However, it is not easy to develop small molecules or antibodies that directly inhibit Fra-1 activity since Fra-1 is a transcription factor with an extended helical structure. An alternative strategy for targeting Fra-1 is to control Fra-1 level instead of Fra-1 activity.

Fra-1 shows a similar expression pattern in other cancer-related genes and is tightly controlled in several ways including transcription, translation, and post-translational modification [15,16]. Since Fra-1 is an intrinsically short-lived protein, stability at the protein level is expected to be modulated by the UPS, as has been shown in c-Fos, another Fos-family member [34]. However, the effect of Fra-1 ubiquitination on stability was not clearly elucidated prior to the current study. Furthermore, the effect of deubiquitination on Fra-1 stability is unknown.

Under this situation, we hypothesized that Fra-1 stability can be controlled by the UPS, and Fra-1 DUB has oncogenic properties by enhancing the Fra-1 target genes involved in cancer metastasis. Accordingly, targeting Fra-1 DUBs can be a promising strategy for mCRC treatment since Fra-1 target genes can be downregulated simultaneously when Fra-1 level is reduced by inhibition of Fra-1 DUBs. To validate this hypothesis, we screened DUBs that ubiquitin-dependently control Fra-1 stability, identified USP21 as a Fra-1 DUB, and confirmed that USP21 overexpression enhances the MMP-1 gene expression transcribed by Fra-1 in colon cancer cells. In parallel, we also confirmed that Fra-1 stability is regulated via the UPS by observing the accumulation of Fra-1 under proteasome inhibitor treatment. Consistent with these observations, Fra-1 is co-localized with USP21 in the cytoplasm, although it has been reported that most Fra-1 is distributed in the nucleus in colorectal cancer [12]. In addition, we show that the stabilizing effect of USP21 on Fra-1 is dependent on the DUB activity of USP21 by observing that mutant USP21 with an inactive catalytic site did not have the DUB effect. Therefore, we propose that the deubiquitinating activity of USP21 regulates nuclear localization of Fra-1.

Furthermore, we demonstrated that USP21 is oncogenic in colorectal cancers by positively regulating Fra-1 activity as a Fra-1 DUB and increasing the expression of MMP-1, the Fra-1 target gene, as summarized in Figure 7D. We demonstrated that USP21 level was highly associated with tumor formation and metastasis both in colon cancer cells and in the animal model, consistent with our findings that CRC tissues highly expressed USP21 and USP21 expression was correlated with tumor progression. Considering that Fra-1 and its AP-1 transcriptional activity are highly elevated in primary tumors and metastases [12], the implication of USP21 in colorectal cancers is consistent with its increase in Fra-1 stability via deubiquitination activity.

Most importantly, we validated the oncogenic property of USP21 in human CRCs by demonstrating that USP21 was highly upregulated in CRC patient samples, especially in high-grade cancers. Additionally, we demonstrated that elevated expression of USP21 is negatively related to survival probability. Our results obtained from molecular and cell biology experiments as well as immunohistochemical assessment of human patient samples were consistent with our mouse model results, namely that USP21 knockdown in colon cancer cells showed reduced metastatic activity.

DUBs have been newly proposed as anti-cancer targets [18], and many DUB inhibitors have been developed as new anti-cancer drugs [35]. Recent studies showed that USP21 is overexpressed in several tumors such as hepatocellular carcinoma [36,37,38]. USP21 was reported to deubiquitinate H2A [39], GATA3 in T cells [40], and Nanog in stem cells [41]. However, USP21-deficient mice were viable and fertile with normal development and morphology. Furthermore, USP21 deficiency had no effect on hematopoiesis or lymphocyte development [42]. Alternatively, Fra-1-knockout mice died in utero likely due to placental defects, and specific deletion of Fra-1 in the mouse embryo resulted in a viable life but with osteopenia, a low-bone mass disease [43]. However, Fra-1-depletion in colon cancer cells showed a defect in formation of metastatic foci in mice [26]. Based on these studies, regulation of USP21 could be an alternative strategy to avoid the side effects derived from depleting substrates. 

AP-1 is known to play an important role in anti-tumor immune responses by inducing the expression of genes for co-inhibitory immune checkpoints and promoting the expression of the master regulator of regulator T cells [44]. Since we demonstrated that USP21 enhances AP-1 target gene expression, combinatorial treatment of immune checkpoint blockers with USP21 knockdown could be beneficial for cancer immunotherapy.

## 4. Materials and Methods

### 4.1. Cell Culture and Reagent

293T (human embryonic kidney), HCT116 (human colon cancer), and HT-29 cells (human colorectal adenocarcinoma) were purchased from the American Type Culture Collection (ATCC, Manassas, VA, USA). 293T and HCT116 cells were cultured in DMEM and HT-29 cells in RPMI1640 medium (Gibco, Los Angeles, CA, USA) supplemented with 10% heat-inactivated fetal bovine serum (FBS) (Gibco) containing penicillin-streptomycin at 37 °C in the presence of 5% CO_2_. Z-Leu-Leu-Leu-CHO (MG132, cat# BML-PI102) was purchased from Enzo Life Science (New York, NY, USA).

### 4.2. Plasmids and Transfection

Full-length human USP21 (NM_001014443.2) was cloned into pDEST-CMV6 with the SRT tag at the N-terminus. The catalytic residue mutant USP21 C221A was generated using PCR mutation. Fra-1 expression plasmid was kindly provided by Jong Hoon Park (Sookmyung Women’s University, Seoul, Korea) and AP-1 reporter plasmids by Jae Youl Cho (Sungkyunkwan University, Seoul, Korea). Plasmids were transfected with TurboFect reagent (Fermentas, Waltham, MA, USA) or jetPRIME^®^ transfection reagent (Illkirch, France) according to the manufacturers’ protocols. 

### 4.3. USP21 Knockdown

For USP21 knockdown, the following USP21 siRNA sequence (NM_001014443.2) was purchased from Bioneer (Daejeon, Korea): 5′-CUGUGAAGCCCUUUAAACA-3′. Small interfering RNAs (siRNAs) were transfected using Lipofectamine^®^ RNAiMAX reagent (Invitrogen, Carlsbad, CA, USA) according to the manufacturer’s protocol. For in vivo experiments, GIPZ Lentiviral Human USP21 shRNA was purchased from Dharmacon (RHS4430, Lafayette, CO, USA).

### 4.4. Reverse Transcription PCR (RT-PCR) and Real-Time PCR (qPCR)

RNA was isolated using Trizol^®^ Reagent (Invitrogen), and cDNA was synthesized with EcoDry™ Premix (Clontech, Mountain View, CA, USA). Quantitative qPCR was performed using the CFX Connect™ Real-Time PCR Detection System (Bio-Rad, Hercules, CA, USA) as previously described [21]. The following primers were used: MMP-1 (Primer Bank, NM_002421), 5′-CTCTGGAGTAATGTCACACCTCT-3′, and 5′-TGTTGGTCCACCTTTCATCTTC-3′; GAPDH (NM_002046.5), 5′-CTGGTAAAGTGGATATTGTTGCCAT-3′, and 5′-TGGAATCATATTGGAACATGTAAACC-3′. 

### 4.5. Luciferase Reporter Assay

Cells were transfected with the indicated plasmids and AP-1 reporter plasmid or human MMP-1 (MMP1-1959luc) promoter luciferase plasmid provided by Jin Ho Chung (Seoul National University, Seoul, Korea) [45]. Renilla luciferase reporter control vector was co-transfected for normalizing luciferase activity using Fermentas transfection reagents. At 48 h after transfection, the cells were collected for dual luciferase reporter assays, and luciferase activity was measured according to the manufacturer’s protocol (Promega, Madison WI, USA). To calculate relative luciferase activity, firefly luciferase activity was divided by Renilla luciferase activity. Data represent the average values from three independent experiments unless otherwise mentioned, and error bars represent standard deviation (SD).

### 4.6. Immunoblotting and Immunoprecipitation (IP)

Cells were solubilized in RIPA lysis buffer (150 mM Tris, pH 7.5, 50 mM NaCl, 1% Triton X-100, 1 mM EGTA, and 10% glycerol) supplemented with protease inhibitor cocktail (Sigma-Aldrich, St. Louis, MO, USA), and immunoblotting was performed with specific antibodies as previously described (Yun et al., 2015). The following primary antibodies were used for immunoblotting: Fra-1 (sc-28310), USP21 (sc-293400 and sc-515911), β-actin (sc-47778) (Santa Cruz Biotechnology, Dallas, TX, USA), and GAPDH (GTX627408, Genetex, Irvine, CA, USA). The antibody against the SRT-tag was made in the animal facility at Sungkyunkwan University School of Medicine. Immunoblotted membranes probed with HRP-conjugated antibody (Bethyl Laboratories, Montgomery, TX, USA) were developed using ECL solution and exposed to X-ray film or analyzed with a luminescent image analyzer (Fujifilm, LAS-3000).

### 4.7. Immunostaining 

Cells were fixed with 3.7% paraformaldehyde for 30 min, permeabilized by 0.1% Triton X-100 in PBS (T-PBS) for 15 min, and blocked with 1% bovine serum albumin (BSA, Sigma-Aldrich) in T-PBS for 30 min. Cells on coverslips were incubated with primary antibodies overnight at 4 °C. After washing with T-PBS, cells were incubated with secondary antibodies at RT for 1 h. Fluorescein isothiocyanate (FITC)-conjugated anti-mouse antibody and Alexa Fluor^®^ 564-conjugated anti-rabbit antibody (Invitrogen) were used for immunodetection. Immunostained cells were analyzed by fluorescence microscopy (ZEISS, Oberkochen, Germany).

### 4.8. Invasion Assay

The invasion assay was performed in a 24-well invasion chamber with an 8 µm pore size polycarbonate membrane according to the manufacturer’s instruction (Millipore, Bedford, MA, USA). Cells (5 × 10^5^) in 300 µL serum-free media were placed into the upper chamber, while the lower chamber was filled with 500 µL media containing 10% FBS. After incubation at 37 °C for 24 h, the invaded cells were stained and counted by microscopic observation. Data represent the average of three individual experiments, and error bars represent the standard error of the mean (SEM).

### 4.9. Migration Assay

Cells were starved in serum-free media for 24 h before being used for migration assay. The migration assay was performed for 16–24 h, according to the manufacturer’s instruction (Millipore). The migrated cells were stained and counted by microscopic observation. Results from triplicate experiments were averaged, and the error bars represent SEM.

### 4.10. Animals, Cells, Intra-Splenic Injections, and Splenectomy in Mice 

Animal experiments were conducted as described in our previous study [46]. BALB/c nu/nu mice (6–8 weeks old, female, *n* = 6 per group) were obtained from the Orient Bio Group (Seoul, Korea) and maintained under specific pathogen-free conditions. All in vivo studies were performed in accordance with institutional guidelines, and the study protocol was approved by the Institutional Animal Care and Use Committee of Samsung Biomedical Research Institute (20170810001). HCT116 cells were transfected with GIPZ shUSP21. After a 24 h incubation, transfected cells were selected with puromycin (Sigma-Aldrich). The selected HCT116-shUSP21 cells were prepared for injection into mice. Nude mice were anesthetized with a mixture of ketamine (100 mg/kg) and Rompun (10 mg/kg) by intraperitoneal injection (0.01 mL/mg) of 3.5 × 10^6^ cells/50 μL Hank’s balanced salt solution (HBSS, Gibco). A small left abdominal flank incision was made, and the spleen was exteriorized for intrasplenic injection. Prepared cells were injected into the spleen with a 30-gauge needle. To prevent cancer cell leakage and bleeding, a cotton swab was held over the injection site for 1 min. Horizon clips were applied under the spleen to occlude the splenic vein, and the dissected wound was sutured with 6-0 black silk. 

### 4.11. In Vivo Tumor Imaging 

After cancer cell injection, MRI imaging (Biospec 7T, Bruker, Billerica, MA, USA) was initiated at day 14 and repeated once a week for as long as liver metastasis was detected. T2-weighted axial MRI sections were obtained using the following settings: fast spin echo sequence with a time to repetition of 1143.8 msec and time to echo of 25.8 ms; 160 × 170 matrix; 24.0 mm × 26.0 mm field of view; signal averaging, 12; section thickness, 1.0 mm; gap, 0 mm. 

### 4.12. Immunohistochemistry (IHC) of In Vivo Tumor

Paraffin-embedded tissue blocks were sectioned at 4 μm thickness, dewaxed in xylene, and then rehydrated in a graded alcohol series. Endogenous peroxidase was blocked with 3% hydrogen peroxide. Tissue sections were immersed in 10 mM citrate buffer (pH 6.0), rinsed in Tris-buffered saline, and then heated in a microwave oven for three 5-min cycles. Sections were stained with hematoxylin and eosin (H&E) to examine tumor morphology before applying the primary antibody. Then, USP21 and Fra-1 were detected with the following primary antibodies: anti-USP21 (ab38864, Abcam, Cambridge, UK) and anti-Fra-1 (SC-28310, Santa Cruz Biotechnology), and sections were subsequently treated with secondary antibody for 30 min and incubated with avidin-biotin-peroxidase complex for 30 min (K5007, Dako, Carpinteria, CA, USA). Diaminobenzidine was used as the chromogen along with slight hematoxylin counterstaining.

### 4.13. Statistics from In Vitro Experiments

Results were normalized to control values and presented as mean ± standard error of the mean. Statistical comparisons between groups were conducted using paired *t*-test or analysis of variance (ANOVA) with multiple comparison tests for post-hoc analysis, unless mentioned otherwise. For *p*-values less than 0.05, differences were considered statistically significant and labeled as such in the corresponding graph.

### 4.14. Collection and Tissue Microarray (TMA) of Human CRC Samples

For this study, tissue specimens from the tumors of 374 patients who had undergone curative surgery for colorectal cancer at Samsung Medical Center, Sungkyunkwan University School of Medicine, Seoul, South Korea were used. TMA was constructed using a manual tissue arrayer (Beecher Instruments, Sun Prairie, WI, USA). Two tissue cores with a diameter of 2.0 mm were obtained from the most representative tumor areas (preferentially, central area and invasive front) of formalin-fixed, paraffin-embedded tissue blocks and were arranged in TMA blocks. 

### 4.15. IHC and Evaluation of CRC Samples 

Sections of the TMA were immunohistochemically labeled with USP21 (Abcam, ab38864, 1:100) using a BenchMark XT automated stainer (Ventana Medical Systems, Inc., Oro Valley, AZ, USA), according to the manufacturer’s protocol. To evaluate expression of USP21, an IHC score was generated by multiplying the percentage of USP21-positive cells by staining intensity. Staining intensity categories were as follows: negative, score of 0; mildly positive, score of 1; moderately positive, score of 2; and strongly positive, score of 3. For example, when all cancer cells were stained with maximal intensity (3+), then the IHC score was 300 (100% × 3). Statistical analyses were performed using SPSS 18.0 statistical software (SPSS). Chi-squared tests (Pearson’s Chi-squared test or Chi-squared test using linear by linear association) were used to analyze correlations between IHC results and clinicopathologic parameters. *p-*Values below 0.05 were considered statistically significant. Survival curves were plotted using the Kaplan–Meier method.

## 5. Conclusions

This study proposes that regulation of Fra-1 by USP21 could be a valuable strategy for mCRC treatment. Furthermore, considering the roles of USP21 and Fra-1 and their abundance in other cancers, USP21 can be considered a potential biomarker and a therapeutic target for various metastatic cancers. Furthermore, combinatorial treatment of drug targeting USP21 can be proposed.

## Figures and Tables

**Table 1:** Demographic and clinicopathological characteristics of studied colorectal cancer (CRC) patients *.

**Oncotype DX**	**Number of Cases**	**IHC Score (** **±SD)**	***p*-Value**
Normal (N)	17	43.5 ± 24.2	vs. AD (*p* = 0.9960722)
Adenoma (AD)	17	42.4 ± 21.7	vs. CA (*p* = 0.0000000)
Carcinoma (CA)	257	121 ± 42.3	vs. N (*p* = 0.0000000)
**Tumor Stage**	**Number of Cases**	**IHC Score (** **±SD)**	***p*-Value**
1	26	107 ± 36.4	vs. 2 (*p* = 0.9162293)
2	92	113 ± 38.3	vs. 3 (*p* = 0.1018557), vs. 4 (*p* = 0.0097765)
3	106	127 ± 42.3	vs. 1 (*p* = 0.1398118), vs. 4 (*p* = 0.3566650)
4	29	141 ± 50.7	vs. 1 (*p* = 0.0148035)
**Lymph Node Metastasis**	**Number of Cases**	**IHC Score (** **±SD)**	***p* Value**
Absent	101	111 ± 38	vs. Low (*p* = 0.0542761)
Low (1~6)	73	127 ± 42.5	vs. High (*p* = 0.1789194)
High (≥7)	27	144 ± 59.1	vs. Absent (*p* = 0.0016502)
**Endolymphatic Emboli**	**Number of Cases**	**IHC Score (** **±SD)**	***p* Value**
Absent	147	117 ± 37.6	vs. Low (*p* = 0.1309999)
Low (rare)	22	136 ± 63.8	vs. Absent (*p* = 0.0000001)
High (frequent)	3	257 ± 11.5	vs. Low (*p* = 0.0000147)

* Immunohistochemistry (IHC) was performed to examine the expression level of USP21 using 297 colorectal cancer (CRC) patient samples. IHC score was calculated by multiplying the percentage of USP21-positive cells by staining intensity on a scale of 0 to 3.

**Table 2:** Tumorigenesis rate from intrasplenic injections.

Group	Cells	Survival Rate	Survival Days	Metastatic Rate		
				Hepatic	Lymphatic	Seeding *
HCT116-control	3.5 × 10^6^	6/6	Day 23 (3 weeks)	6/6 (100%)	0/6	1/6 (16.67%)
HCT116-shUSP21	3.5 × 10^6^	** 4/6	Day 23 (3 weeks)	4/4 (100%)	0/4	0/4

* “Seeding” represents the formation of an intraperitoneal tumor which is considered to be caused by leakage of tumor cells during intrasplenic injection and splenectomy. ** Two of six animals unexpectedly died on day 7 and 21, respectively.

## Supplementary Materials

The following are available online at https://www.mdpi.com/2072-6694/12/1/207/s1, **Table S1**: Recurrence-free survival of CRC patients, Table S2: Overall survival of CRC patients, Figure S1: (**A**) Effect of SRT-USPs on Fra-1 mediated AP-1 transcriptional activity. (**B**) Effect of several selected USPs on protein stability of Fra-1, Figure S2: Knockdown effects of siUSP21 sequences, Figure S3: Effects of SRT-USP21 (wild type and C221A mutant) on Fra-1 mediated activity, Figure S4: Effect of the USP21 overexpression on the Fra-1 mRNA expression, Figure S5: shUSP21 colony selection in HCT116 cells. Immunoblot images.
