# Supplementary material for: Ubiquitin-Specific Protease 21 Promotes Colorectal Cancer Metastasis by Acting as a Fra-1 Deubiquitinase
Source: Cancers (Basel). 2020 Jan 14;12(1):207. doi: 10.3390/cancers12010207 (PMC7017141; doi:10.3390/cancers12010207)
Supplement: Supplementary file 1 [file cancers-12-00207-s001.pdf]

Supplementary Materials

Ubiquitin Specific Protease 21 Promotes Colorectal Cancer Metastasis by Working as a Fra-1 Deubiquitinase

Sun-Il Yun, Hye Kyung Hong, So-Young Yeo, Seok-Hyung Kim, Yong Beom Cho and Kyeong Kyu Kim

Supplementary Tables

Table S1. Recurrence-free survival of CRC patients.

Group	Cases *			
	0 Month	50 Months	100 Months	150 Months
USP21 negative	39	29	25	0
USP21 positive	203	111	71	1

* Recurrence-free survival cases are classified according to the survival months. Number of cases in each group and their correlation with the USP21 expression are listed. This result is plotted as Kaplan-Meier survival curves in Figure 6E.

Table S2. Overall survival of CRC patients.

Group	Cases *			
	0 Month	50 Months	100 Months	150 Months
USP21 negative	39	32	29	0
USP21 positive	202	127	102	1

* Overall survival cases are classified according to the survival months. Number of cases in each group and their correlation with the USP21 expression are listed. This result is plotted as Kaplan-Meier survival curves in Figure 6F.

Supplementary Figures

A

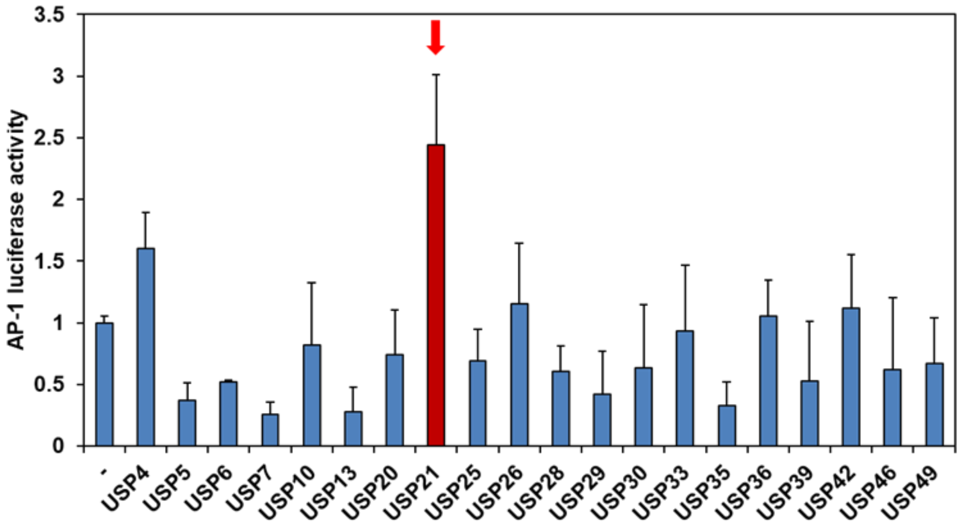

B

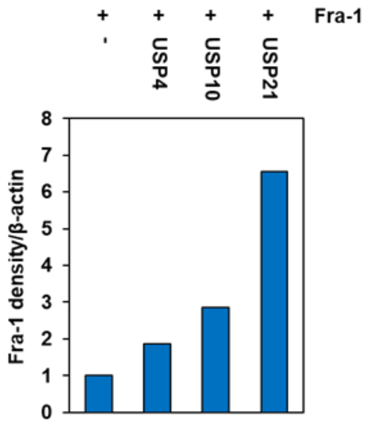

Figure S1. (**A**) Effect of SRT-USPs on Fra-1 mediated AP-1 transcriptional activity. 293T cells were co-transfected with selected USPs and Fra-1 expression plasmids along with AP-1 reporter plasmid. Genes coding full-length human USPs were cloned into pDEST-CMV6 with SRT tag at the N-terminus. Luciferase transcriptional response induced by Fra-1 was measured in 293T cells. Data show the relative ratio compared to Fra-1 alone and are presented as means ±SD. (**B**) Effect of several selected USPs on protein stability of Fra-1. USPs were detected with anti-SRT antibody: USP4, USP10, and USP21 (left). The density of Fra-1 band was quantified with normalization with respect to a loading control (β-actin) and plotted (right).

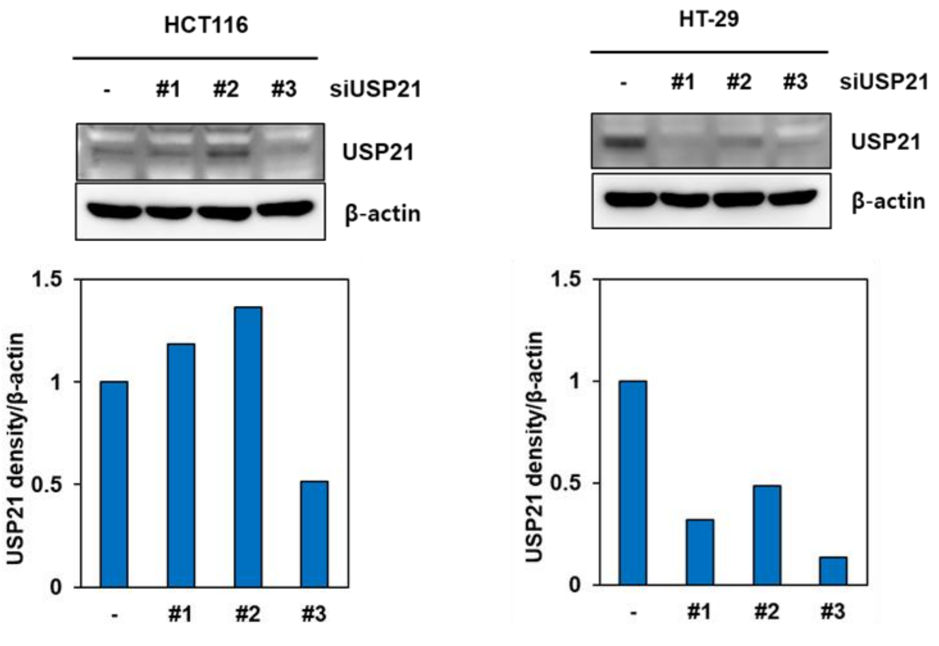

Figure S2. Knockdown effects of siUSP21 sequences. To evaluate knockdown effect of siUSP21 sequences, HCT116 (left) and HT29 cells (right) were co-transfected with three different siRNA sequences and USP21 protein expression was examined by immunoblotting. The density of USP21 band was quantified and normalized by β-actin. From this result, the third siRNA (#3) of USP21 was selected and used in this study.

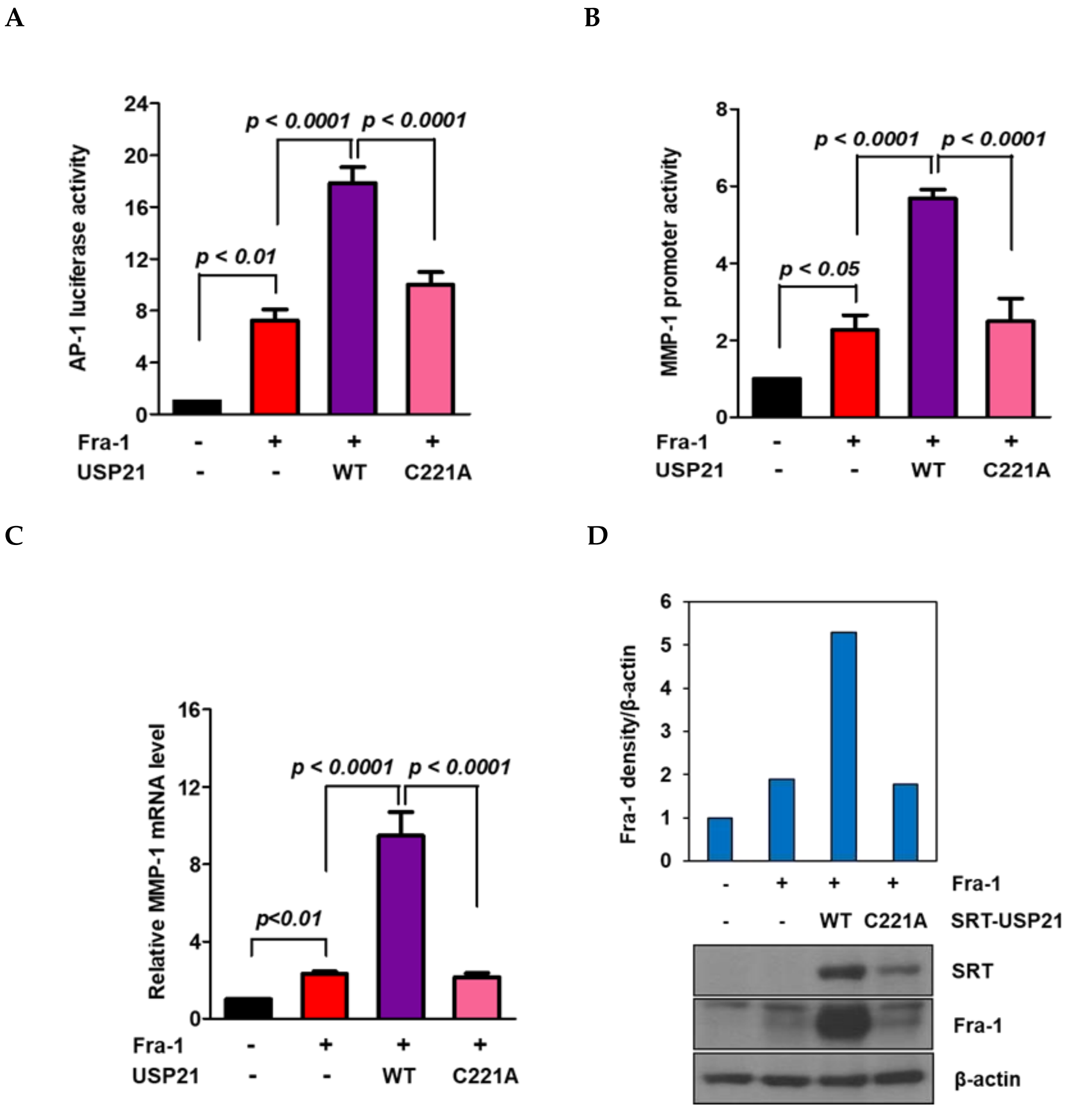

Figure S3. Effects of SRT-USP21 (wild type and C221A mutant) on Fra-1 transcriptional activity. Fra-1 expression plasmid was co-transfected with SRT-USP21 (wild type and C221A mutant) expression vectors, along with AP-1-luciferase in 293T cells (**A**) or MMP-1 promoter plasmids in HCT116 cells (**B**). Luciferase transcriptional response induced by Fra-1 was measured in each cell line. Data are presented as means ±SD. (**C**) MMP-1 mRNA level was determined in HCT116 cells transfected with Fra-1 and SRT-USP21 WT or SRT-USP21 C221A for 24 h. Relative expression of each mRNA was quantified by real time-PCR with normalization to GAPDH. Values are fold-increases relative to the control of three independent experiments, and error bars represent means ±SD. Data were statistically analyzed by one-way ANOVA (n = 3, A–C). (**D**) Transfection efficiency of USP21 for 24h in HCT116 cells was analyzed by immunoblot analysis with indicated antibodies and shown with a representative data. β-actin was used as a loading control. The band intensity of Fra-1 was quantified with normalizing with respect to β-actin and shown as a graph.

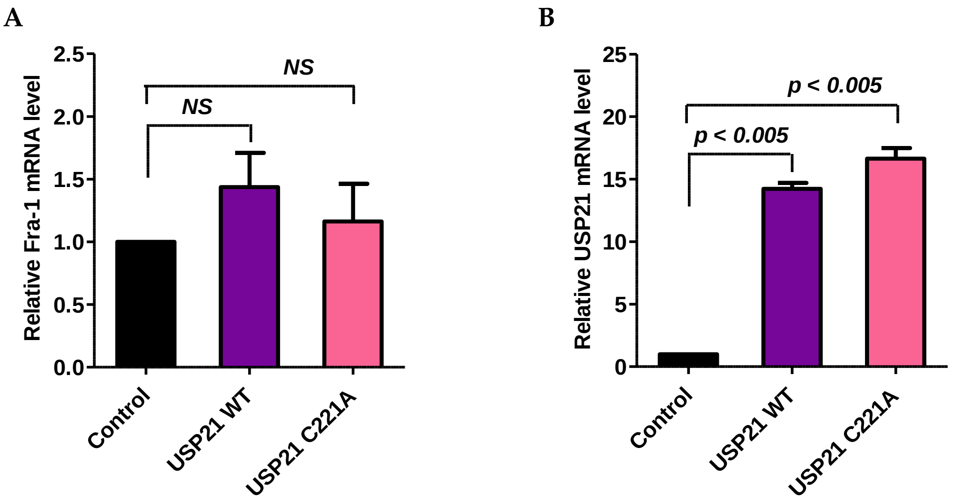

Figure S4. Effect of the USP21 overexpression on the Fra-1 mRNA expression. To evaluate the effect of overexpression of USP21 on Fra-1 mRNA expression, HCT116 cells were transfected with plasmids encoding USP21 WT or USP21 C221A. The mRNA expression of Fra-1 (**A**) and USP21 (**B**) was quantified by real time-PCR with normalization to GAPDH. The data were statistically analyzed by one-way ANOVA ($n = 3$). NS is no significant.

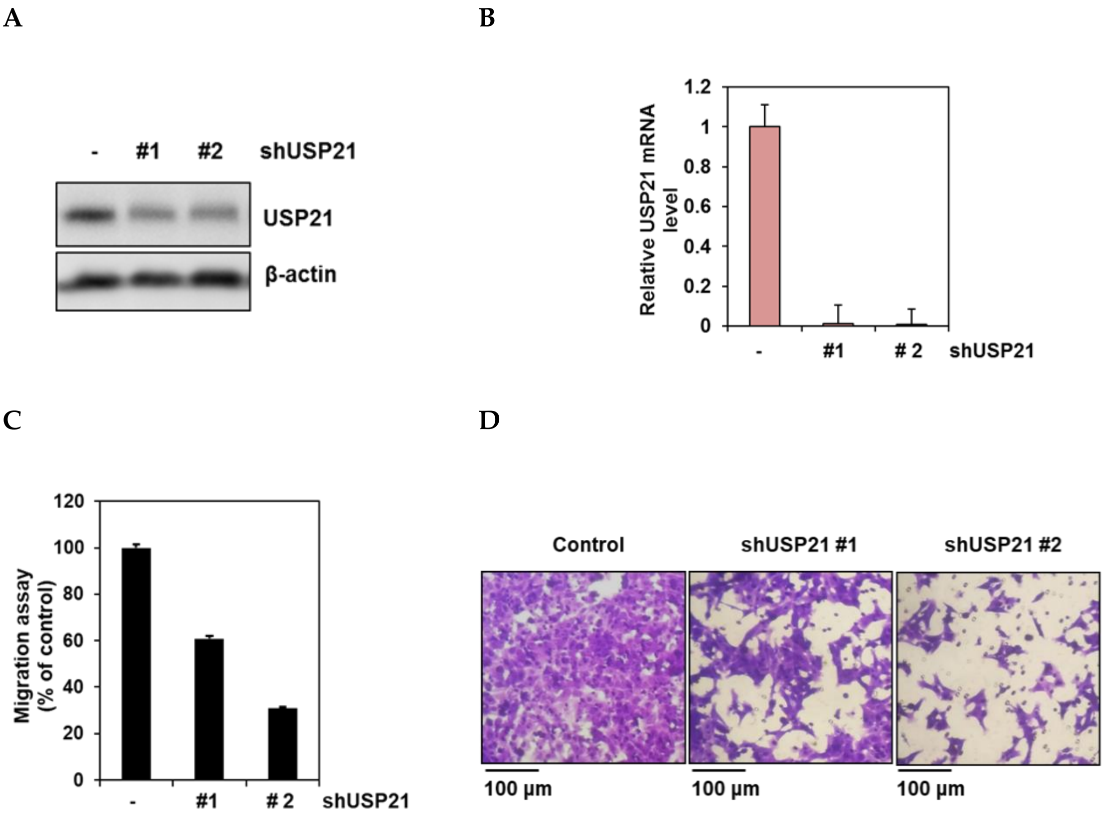

Figure S5. shUSP21 colony selection in HCT116 cells. HCT116 cells were transfected with shUSP21 for 24 h and then treated with puromycin for 3 days. Selected colony was lysed and protein expression of USP21 was evaluated by immunoblotting with specific antibody (**A**). USP21 mRNA level was quantified from two representative colonies (**B**). Cell migration activity of the selected colonies was measured by transwell assay. Number of migrated control cells was set to 100% and that of shUSP21 cells was shown by the % ratio (**C**). Representative images of migrated cells were shown at right (scale bar = 100 μm) (**D**).

Immunoblot Images

Figure 1A

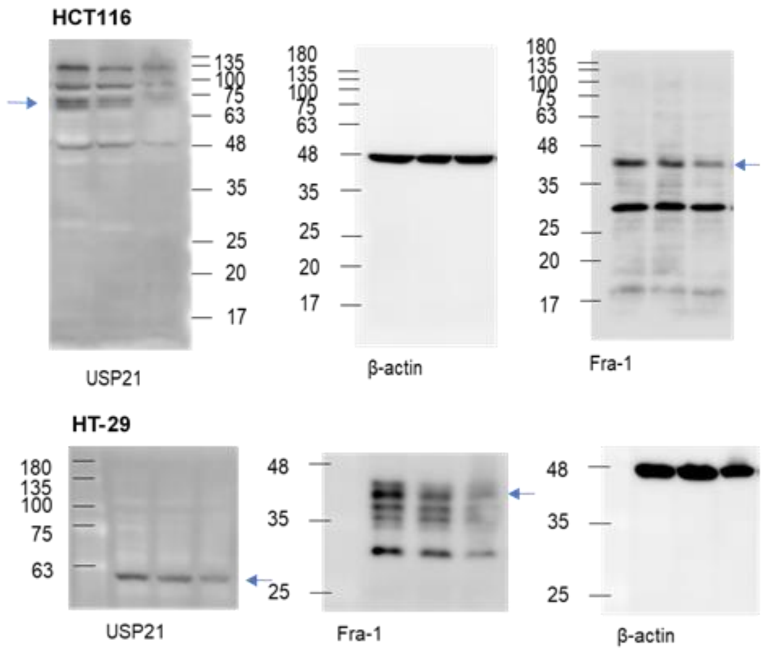

Figure 1B, left

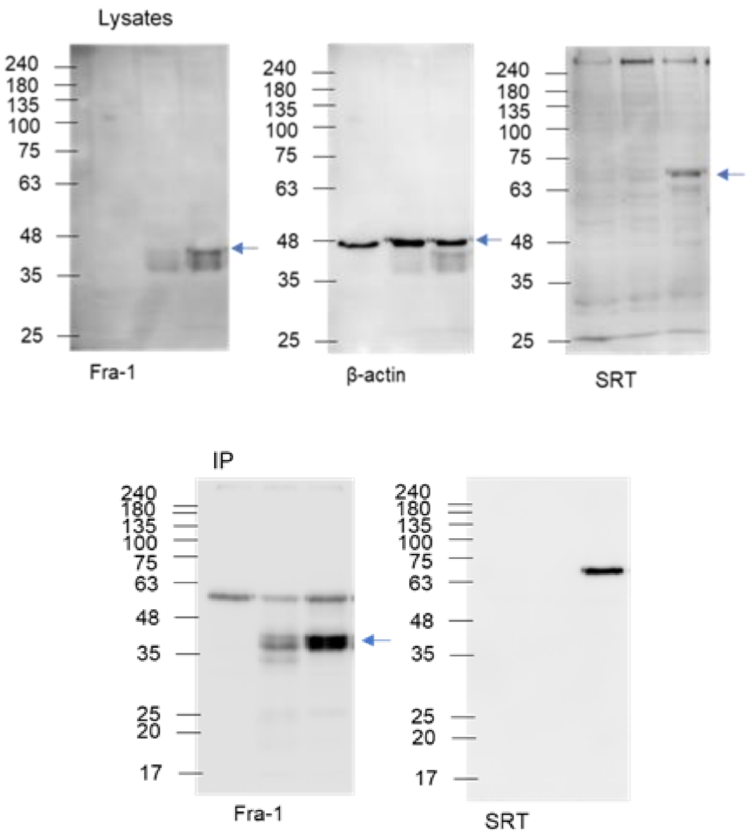

Figure 1B, right

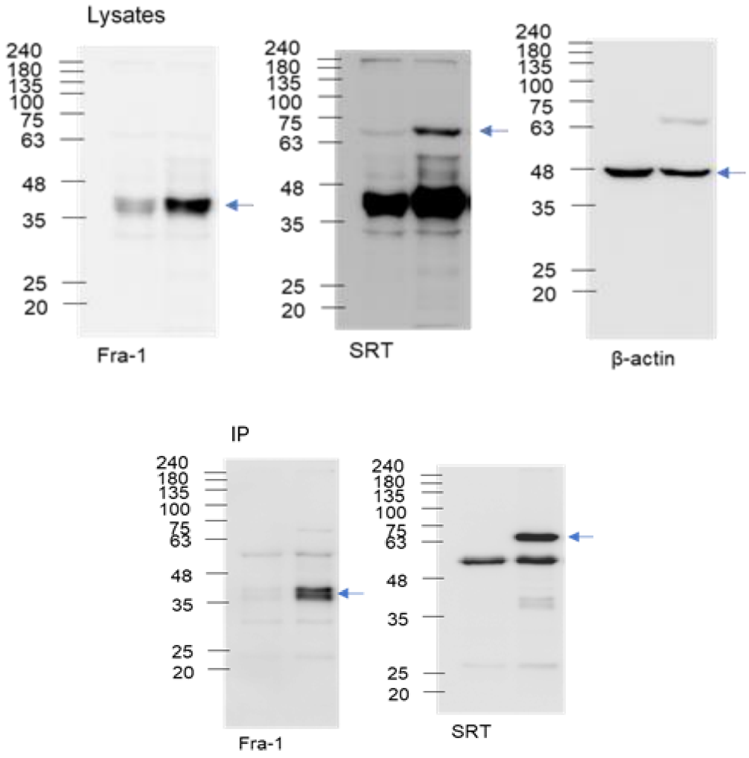

Figure 2A

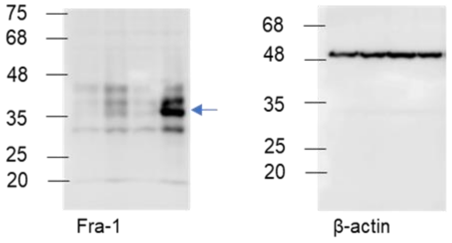

Figure 2B

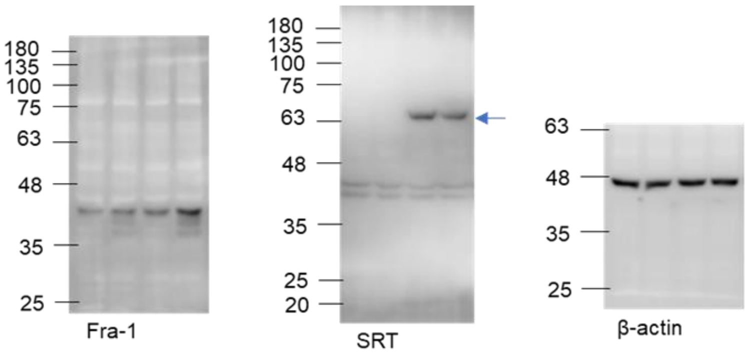

Figure 3A

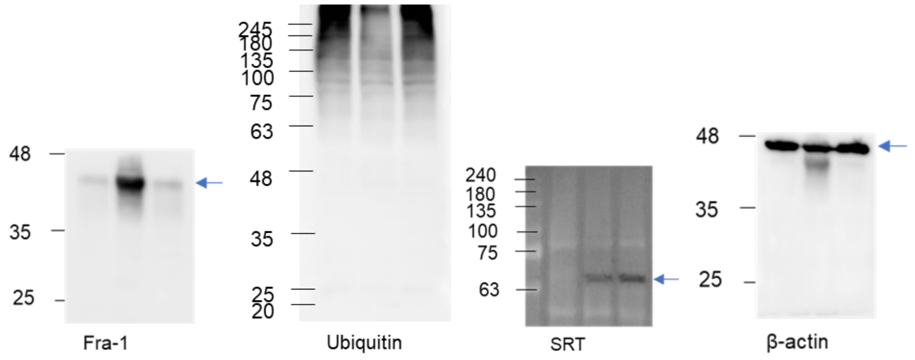

Figure 3B

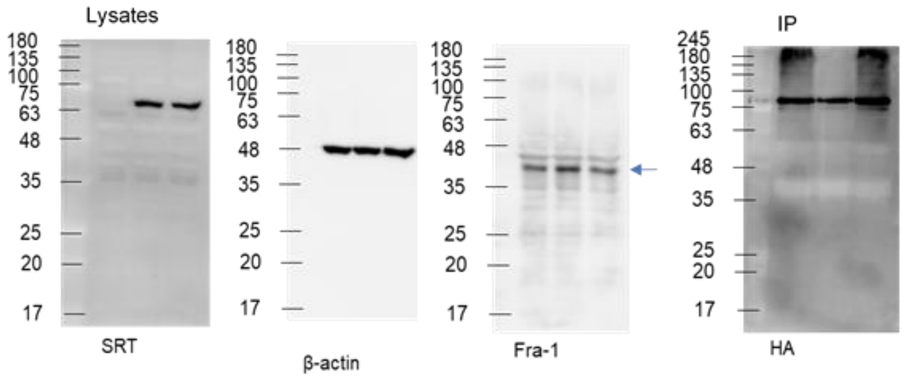

Figure 3C

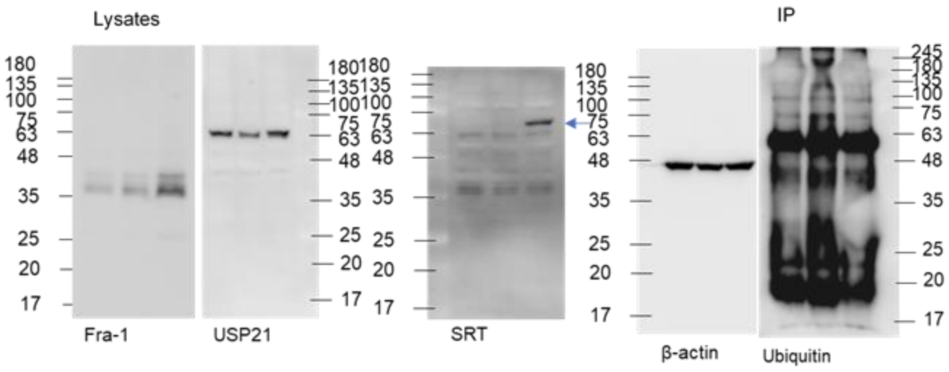

Figure 3D

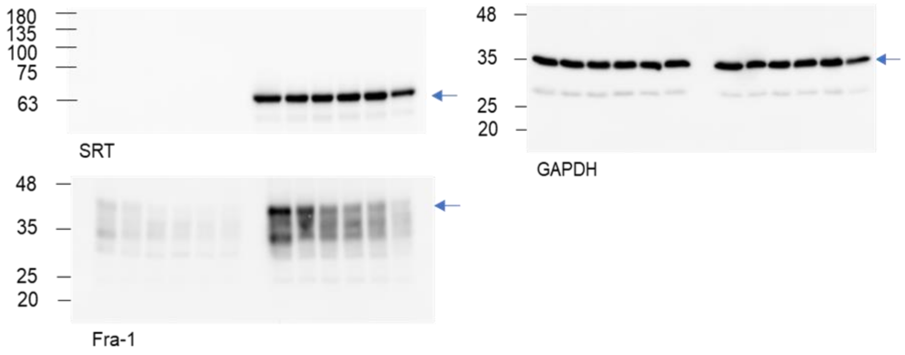

Figure 4D

HCT116

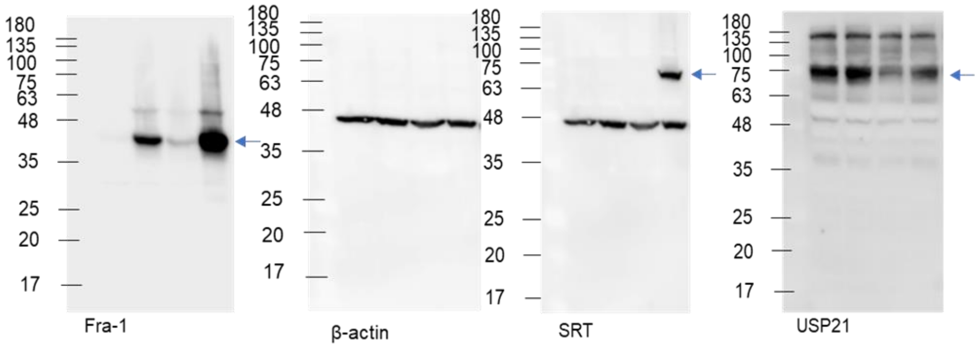

HT29

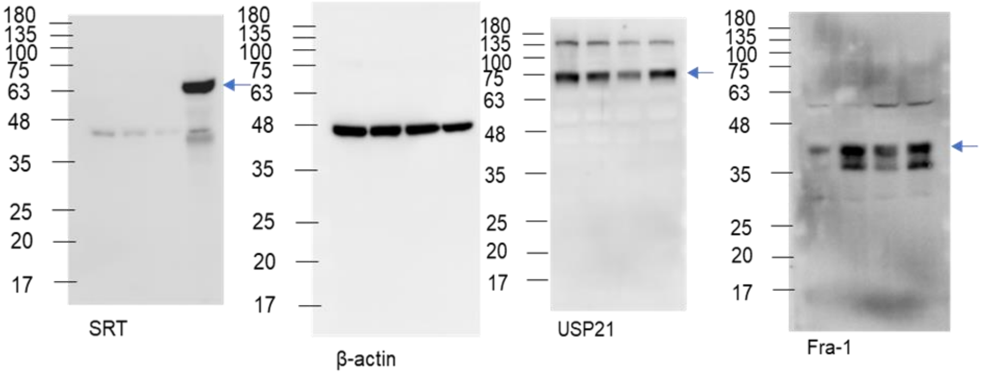

Figure 5C

HCT116

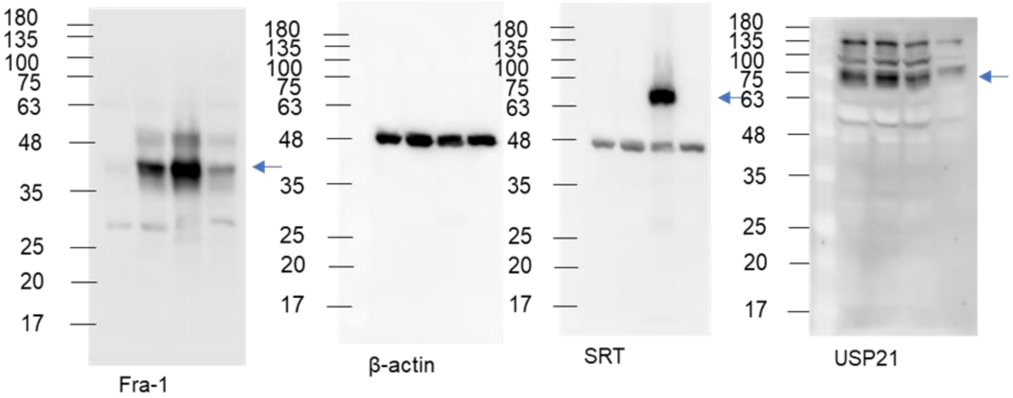

HT-29

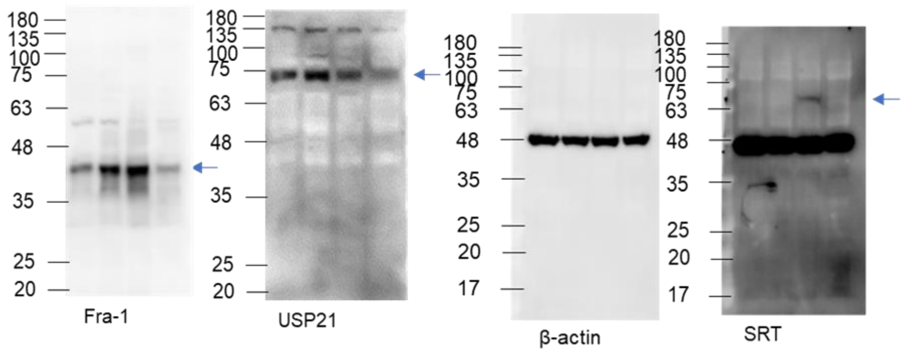

Supplementary Figure S1.

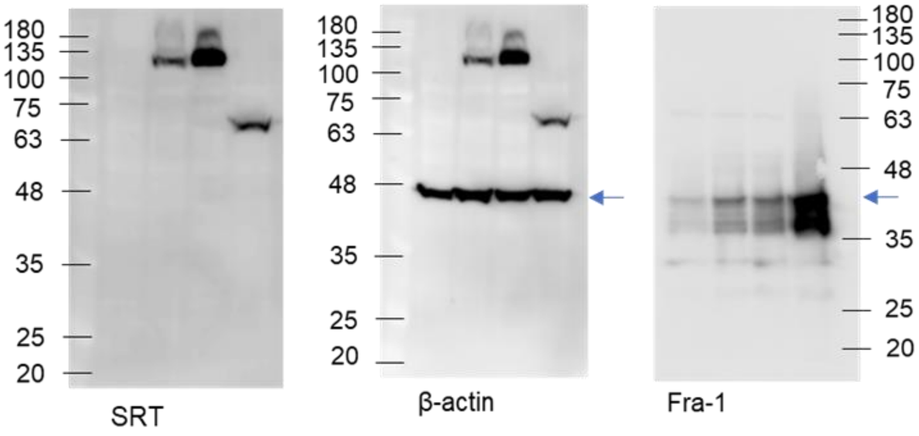

Supplementary Figure S2

HCT116 **HT29**

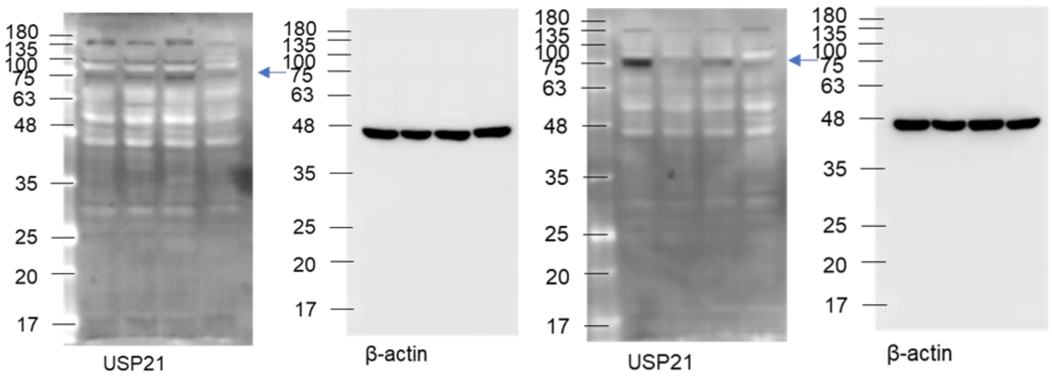

Supplementary Figure S3

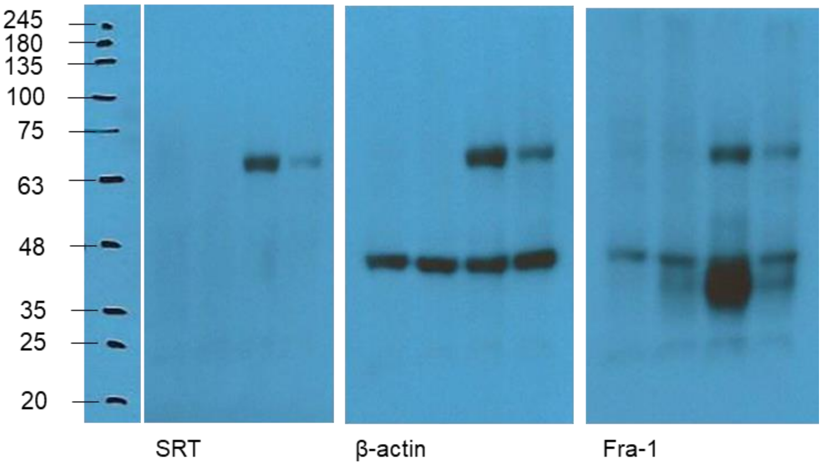

Supplementary Figure S3

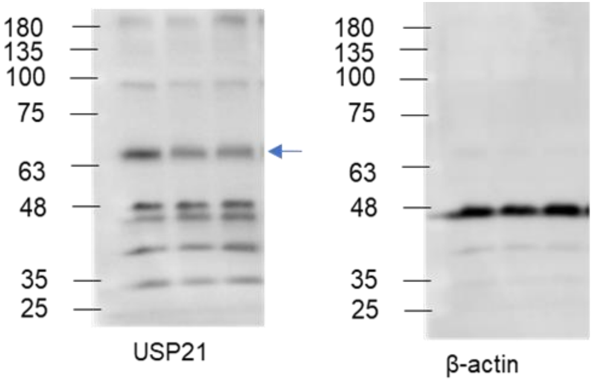

© 2020 by the authors. Licensee MDPI, Basel, Switzerland. This article is an open access article distributed under the terms and conditions of the Creative Commons Attribution (CC BY) license (http://creativecommons.org/licenses/by/4.0/).
